# Supplementary material for: MHC-I upregulation by macbecin II in the solid tumors potentiates the effect of active immunotherapy
Source: EMBO Mol Med. 2025 Mar 14;17(4):797–822. doi: 10.1038/s44321-025-00213-7 (PMC11982318; doi:10.1038/s44321-025-00213-7)
Supplement: Supplementary file 1 — Table EV1 [file 44321_2025_213_MOESM1_ESM.docx]

**Table EV1:** List of primers used.

| Gene | Primer sequence (5'-3') |
| --- | --- |
| GAPDH(H) F | GGTCGGAGTCAACGGATTTG |
| GAPDH (H) R | GACAAGCTTCCCGTTCTCAG |
| GAPDH (M) F | GGTCGGTGTGAACGGATTTG |
| GAPDH (M) R | GCCGTTGAATTTGCCGTGAG |
| MHC I (H) F | GGCTACGTGGACGACACGCAG |
| MHC I (H) R | GCCTTCACATTCCGTGTCTC |
| MHC II (H) F | GAGCCCACAGTGACCATCT |
| MHC II (H) R | CCAGGATCTGGAAAGTCCAGT |
| H2K F | CCCACACTCGCTGAGGTATT |
| H2K R | CCAATACTCGGGCCCCTC |
