# Supplementary figures and images for: MHC-I upregulation by macbecin II in the solid tumors potentiates the effect of active immunotherapy
Source: EMBO Mol Med. 2025 Mar 14;17(4):797–822. doi: 10.1038/s44321-025-00213-7 (PMC11982318; doi:10.1038/s44321-025-00213-7)

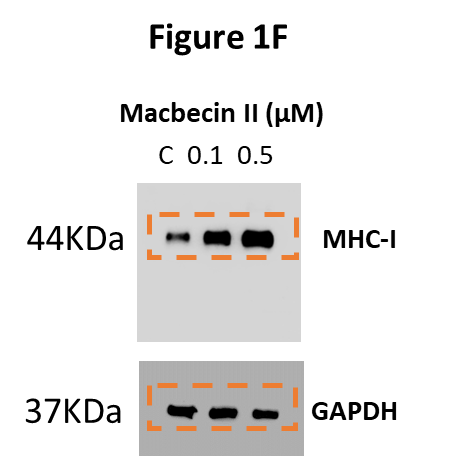


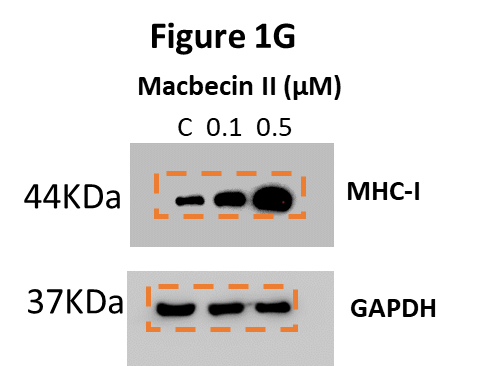

Supplement: Supplementary file 3 — Source data Fig. 1 [file 44321_2025_213_MOESM3_ESM.zip › Figure 1 WB.docx]

Figure 4C


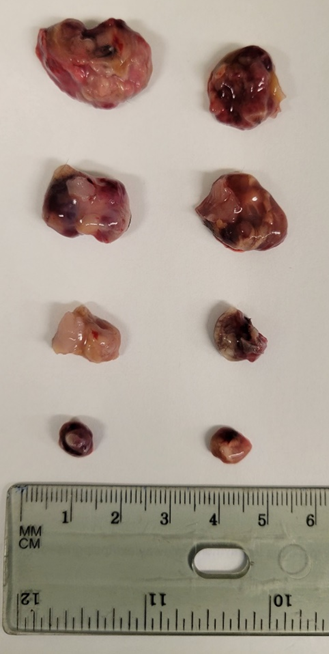


Figure 4L


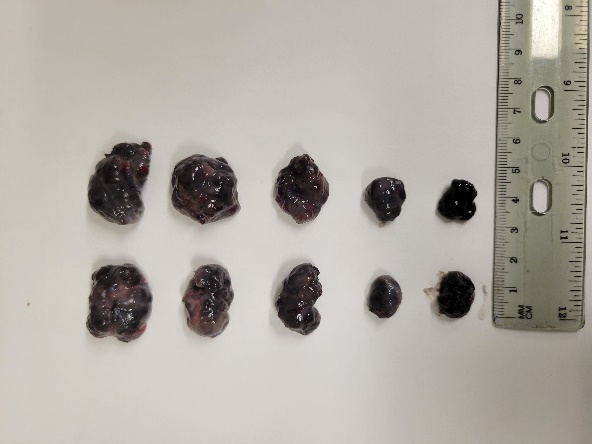


Figure 4E


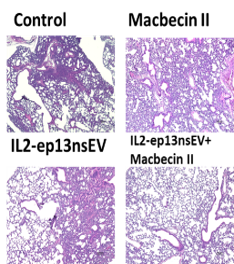


Figure 4N


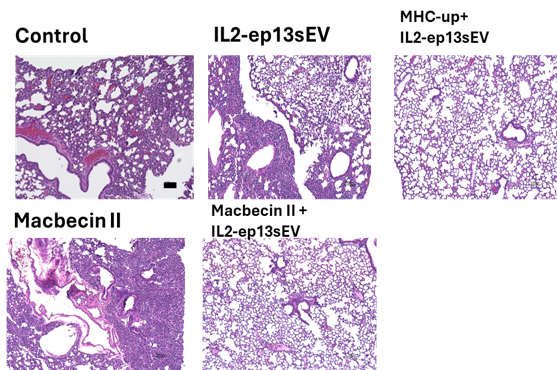

Supplement: Supplementary file 6 — Source data Fig. 4 [file 44321_2025_213_MOESM6_ESM.zip › Figure 4 Whole mount and image.docx]

Figure 5B


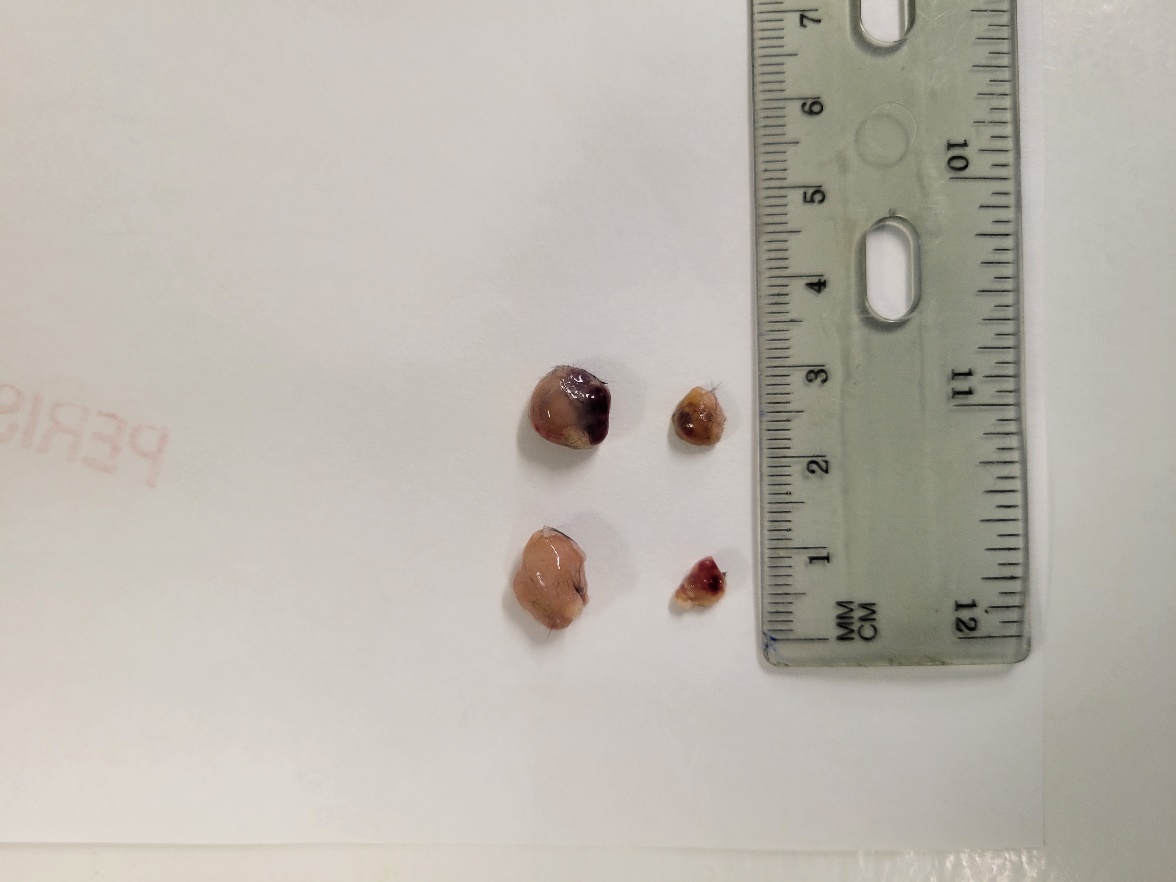


Figure 5D


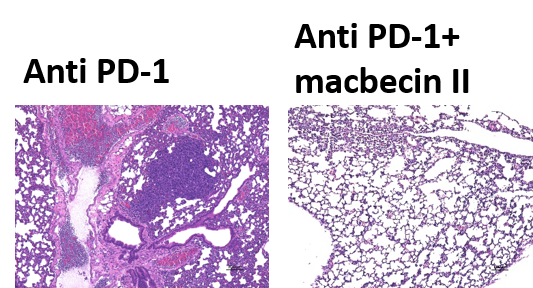


5E


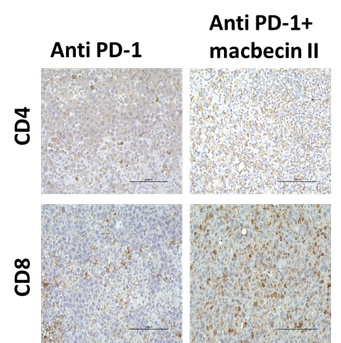


5F


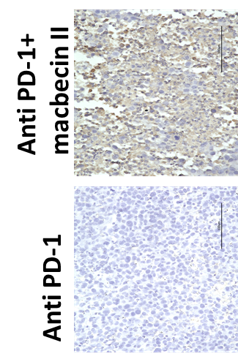

Supplement: Supplementary file 7 — Source data Fig. 5 [file 44321_2025_213_MOESM7_ESM.zip › Figure 5 whole mount and images.docx]

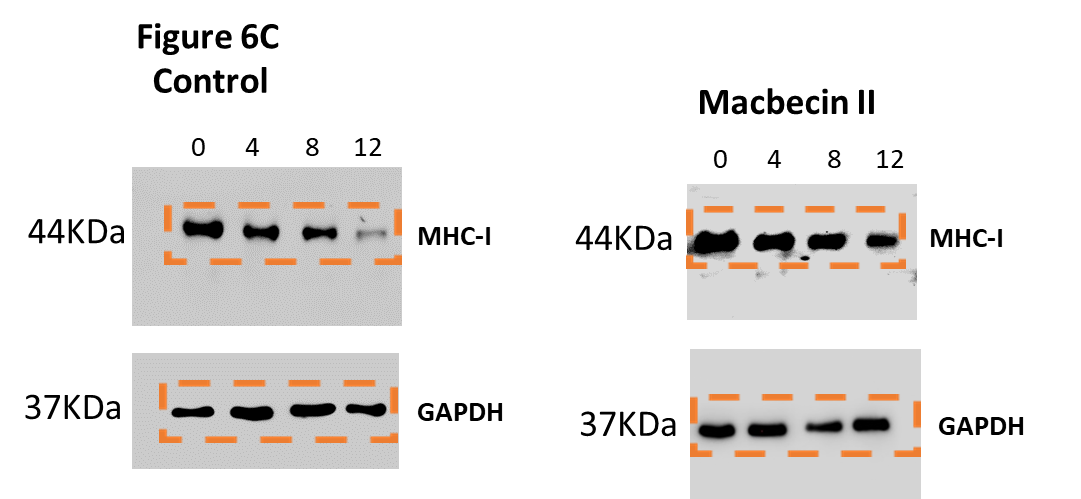


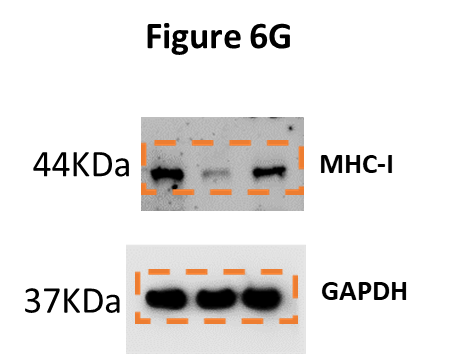


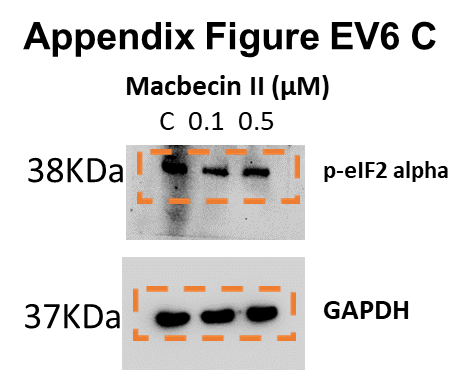


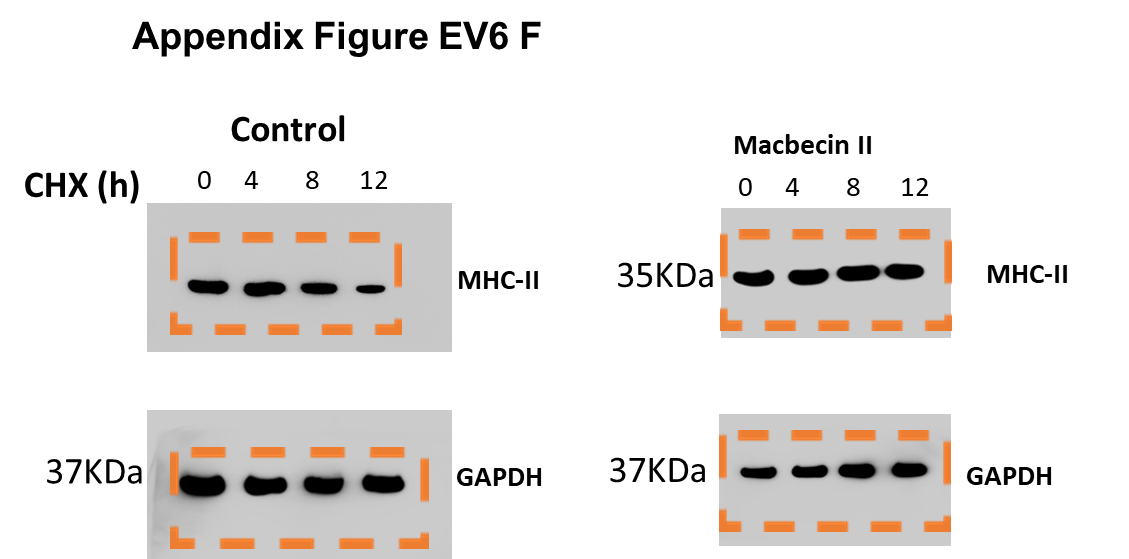


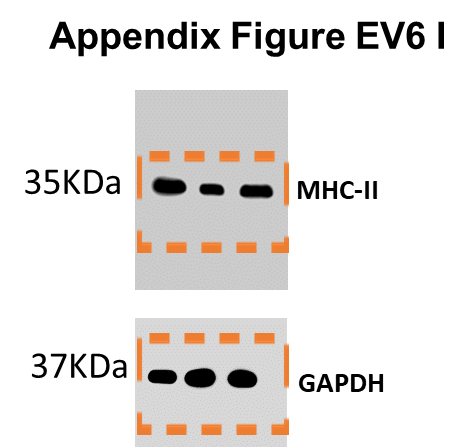


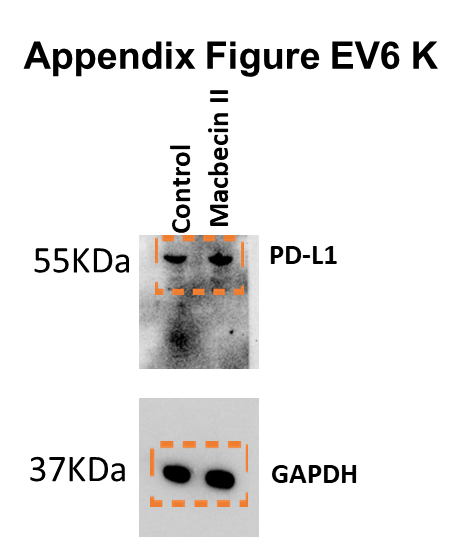

Supplement: Supplementary file 8 — Source data Fig. 6 [file 44321_2025_213_MOESM8_ESM.zip › Figure 6.docx]
