# Supplementary material for: MHC-I upregulation by macbecin II in the solid tumors potentiates the effect of active immunotherapy
Source: EMBO Mol Med. 2025 Mar 14;17(4):797–822. doi: 10.1038/s44321-025-00213-7 (PMC11982318; doi:10.1038/s44321-025-00213-7)
Supplement: Supplementary file 9 — Expanded View Figures [file 44321_2025_213_MOESM9_ESM.pdf]

Expanded View Figures

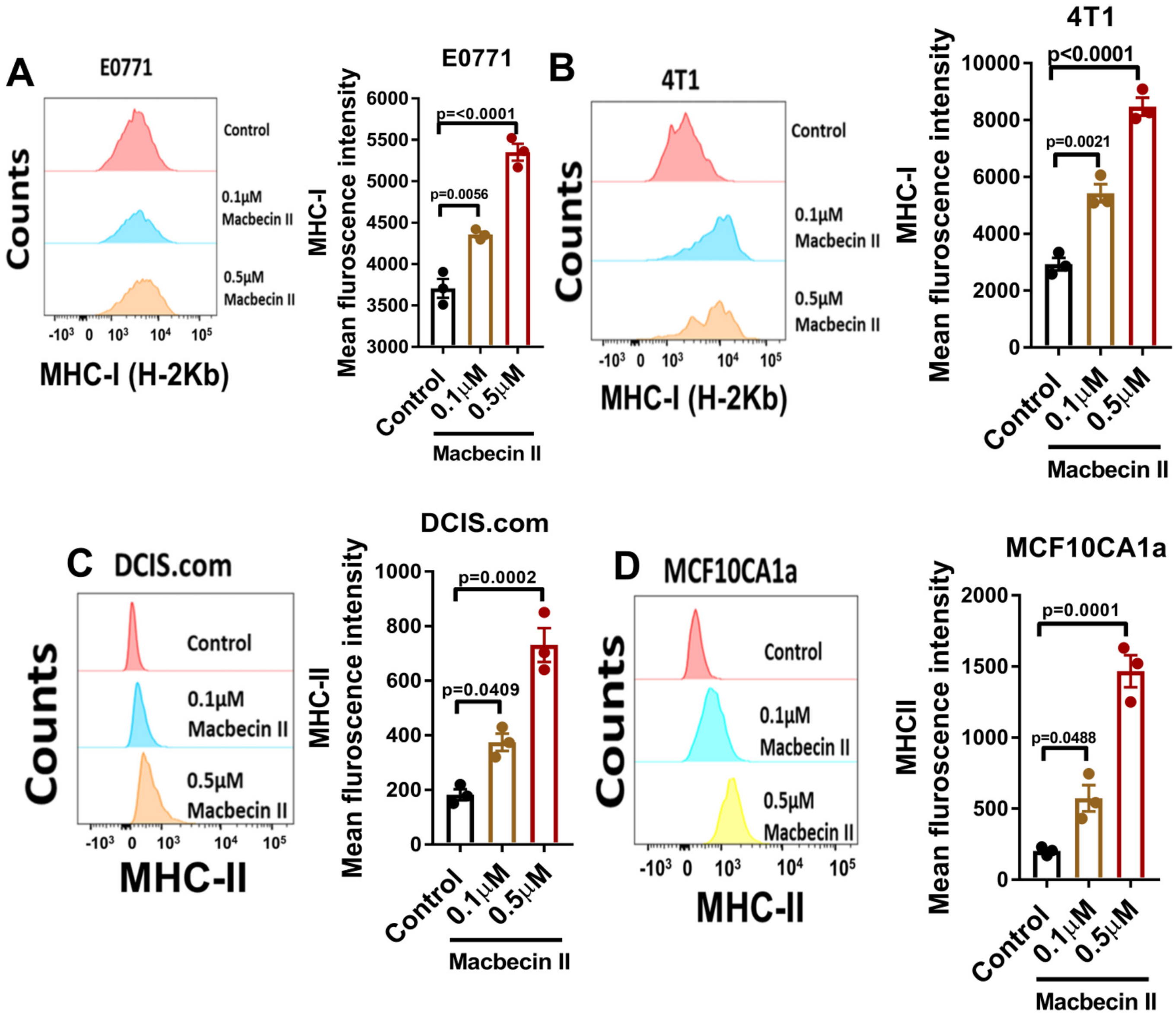

Figure EV1. Macbecin II stimulates MHC-I and II expression.

(**A**) E0771 cells were seeded in a 24-well plate and treated with the indicated dose of macbecin II for 48 h. Cells were trypsinized and stained with anti-MHC I (H-2Kb) primary and secondary antibody. The readouts were obtained by BD FACS Canto. Mean fluorescence intensity was calculated and compared by one-way ANOVA with a Tukey post-hoc test ($n = 3$/group, biological replicates). (**B**) 4T1 cells were treated with the indicated dose of macbecin II for 48 h and MHC-I (H-2Kb)expression was examined as described in (**A**). Mean fluorescence intensity was calculated and compared by one-way ANOVA with a Tukey post-hoc test ($n = 3$/group, biological replicates). (**C**) DCIS.com cells were treated with macbecin II at the indicated doses for 48 h. The cells were trypsinized and stained with anti-MHC-II primary followed by secondary antibodies. Readouts were obtained using a BD FACS Canto. Mean fluorescence intensity was calculated and analyzed using the one-way ANOVA with a Tukey post-hoc test ($n = 3$/group, biological replicates). (**D**) MCF10CA1a cells were treated with macbecin II at the indicated doses for 48 h. The cells were processed as described in (**C**), and the results were analyzed using the one-way ANOVA with a Tukey post-hoc test ($n = 3$/group, biological replicates). Data are represented as mean +/− SEM.

 © The Author(s)

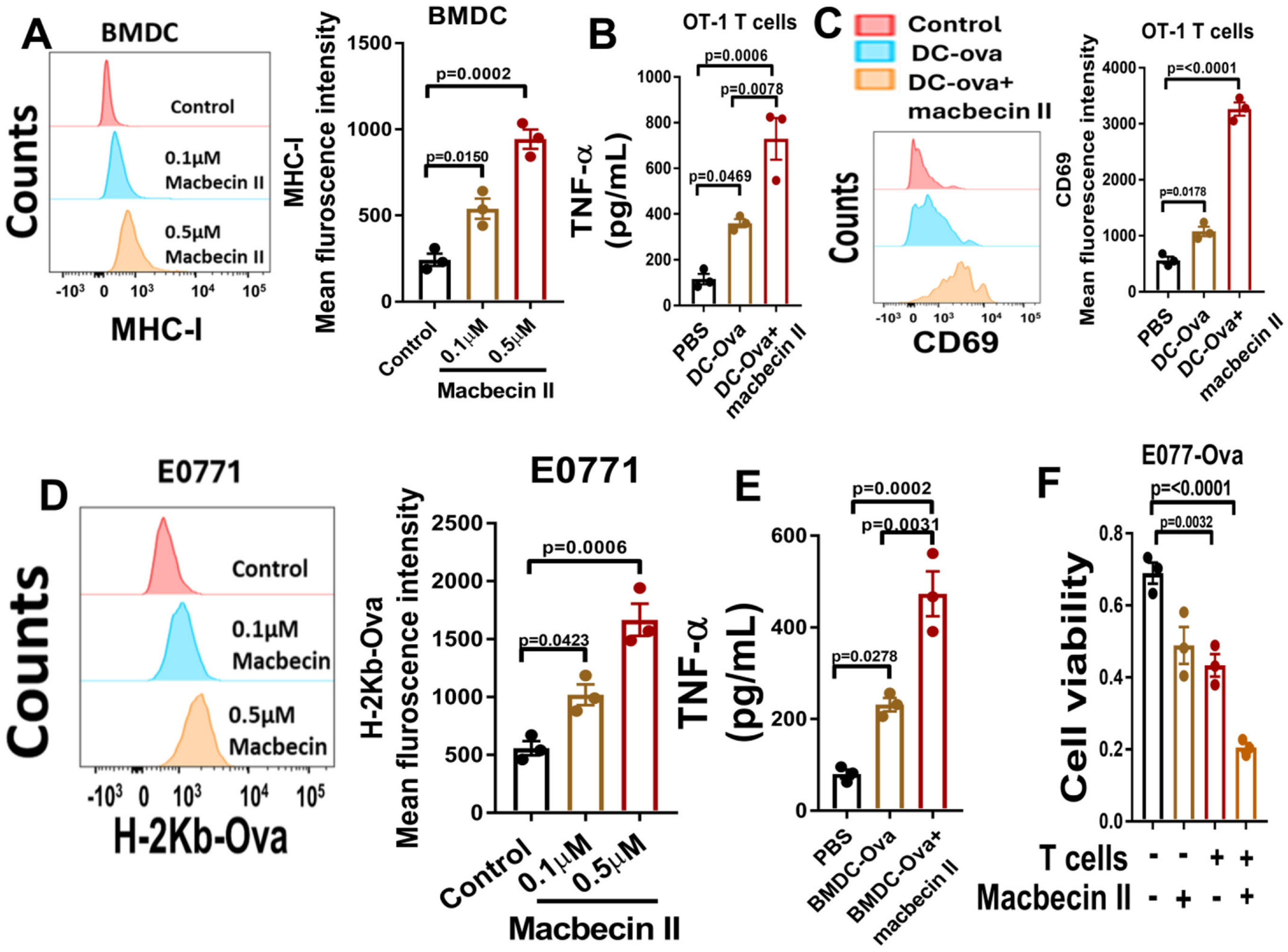

Figure EV2. Macbecin II promotes antigen presentation and enhances antigen-dependent cancer cell death.

(**A**) BMDCs were treated with the indicated dose of macbecin II for 48 h. Post-incubation, cells were stained with an H-2Kb antibody and examined by FACS ($n = 3$/group, biological replicates). Statistical inference was compared by one-way ANOVA with a Tukey post-hoc test. (**B**) OT-1 cells were co-cultured with B16-Ova cells and the expression of TNF-α in the medium was measured by ELISA. Statistical significance was determined using one-way ANOVA with a Tukey post-hoc test ($n = 3$/group, biological replicates). (**C**) OT-1 T cells were co-cultured in the presence of DCs that were pulsed with Ova (DC-ova) with or without macbecin II, and then examined for CD69 surface expression by FACS at after three days. Statistical significance was determined using the one-way ANOVA with a Tukey post-hoc test ($n = 3$/group, biological replicates). (**D**) E0771 cells transfected with the ova plasmid were treated with the indicated doses of macbecin II for 48 h. The cells were examined for the presentation of H-2Kb Ova peptide by FACS. One-way ANOVA with a Tukey post-hoc test was used for statistical analysis ($n = 3$/group, biological replicates). (**E**) The BMDCs were isolated from the syngeneic mouse and pulsed with ova peptide with or without macbecin II. TNF-α levels were examined in T cells co-cultured with BMDCs ($n = 3$/group, biological replicates). (**F**) E0771-Ova cells (500 cells/well) were seeded in a 96-well plate and treated with the indicated doses of macbecin II followed by co-culturing with T cells (E:T ratio 10:1) from (**E**) for 48 h. Dead cells were washed out with ice-cold PBS, and the cancer cells were fixed with methanol. and stained with crystal violet. The dye was then dissolved in 10% acetic acid, and readouts were obtained at 590 nm. Statistical significance between groups was determined using the one-way ANOVA with a Tukey post-hoc test ($n = 3$/group, biological replicates). Data are presented as mean +/− SEM.

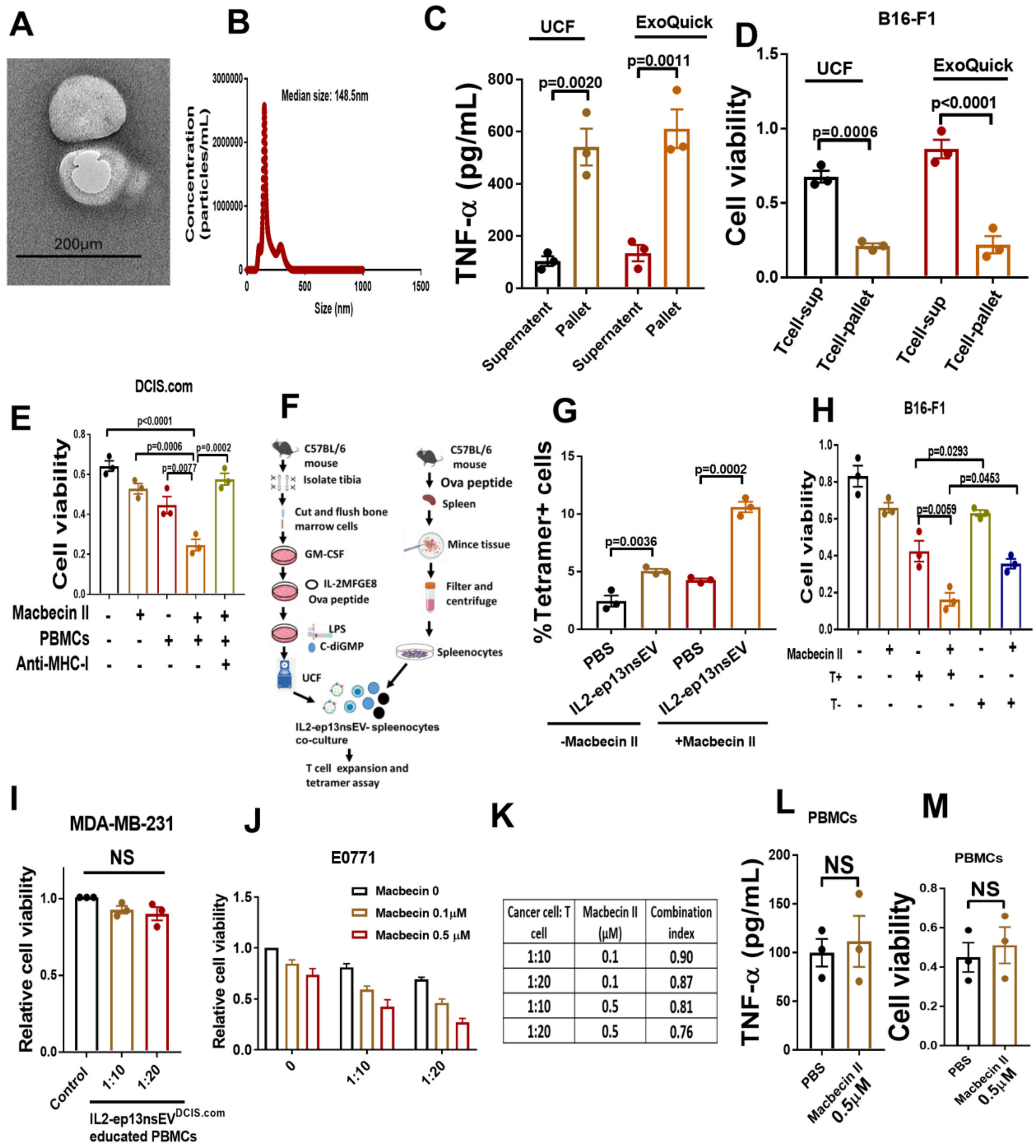

Cancer cell: T cell	Macbecin II (μM)	Combination index
1:10	0.1	0.90
1:20	0.1	0.87
1:10	0.5	0.81
1:20	0.5	0.76

Figure EV3. Macbecin II potentiates anticancer efficacy of IL2-ep13nsEV in vitro.

(**A**) Extracellular vesicles were isolated from dendritic cells that were pulsed with DCIS.com lysate. They were examined by electron microscopy. Representative image is shown. (**B**) The size distribution of p13nsEV was examined by Nanoparticle tracking analysis (NTA). The median size of p13nsEV was 148.5 nm. (**C**) The exosomes were isolated from the BMDCs pulsed with B16-F1 lysate by ultracentrifugation (UCF) or with the use of ExoQuick kit. The pallet or the supernatant fraction isolated from the UCF was then incubated with the T cells isolated from the spleen. Similarly, exosome fraction or exosome-depleted fraction from ExoQuick preparation was incubated with the T cells. The T cells were then co-cultured with the B16-F1 cells and TNF-α was examined ($n = 3$/group, biological replicates). The result was analyzed by the unpaired two-tailed Student's t-test ($n = 3$/group, biological replicates). (**D**) The T cells treated with the supernatant and pallet fraction were co-cultured with the B16-F1 cells (E:T ratio 15:1) for 48 h and relative cell viability was examined. After the incubation, the cells were washed with ice-cold PBS to remove dead cells. The live cells were fixed with methanol at room temperature for 15 min and then stained with crystal violet. The dye was dissolved in 10% acetic acid, and readouts were obtained at 590 nm. The result was analyzed by the unpaired two-tailed Student's t-test ($n = 3$/group, biological replicates). (**E**) DCIS.com cells (500 cells/well, $n = 3$/group, biological replicates) were seeded in a 96-well plate and co-cultured in the presence of PBMCs, macbecin II (0.1 μM), or 40 μg/mL MHC-I blocking antibody for 48 h. The IL2-ep13nsEV were prepared from the dendritic cells that were pulsed with DCIS.com lysate and they were used to educate T cells in the PBMC. After the incubation, the cells were washed with ice-cold PBS to remove dead cells. The live cells were fixed with methanol at room temperature for 15 min and then stained with crystal violet. The dye was dissolved in 10% acetic acid, and readouts were obtained at 590 nm. Statistical significance was determined using the one-way ANOVA with a Tukey post-hoc test. (**F**) Schematic of the experiment. BMDC were treated with GM-CSF for 5 days to differentiate monocytes into dendritic cells (DCs). The DCs were treated with ova peptide, LPS (100 ng/ml), C-diGMP (200 μM), and macbecin II followed by IL2-ep13nsEV isolation. The T cells were isolated from the spleen of syngeneic mice that were subcutaneously injected with ova peptide (5 μg for 10 days), with or without macbecin II, and they were activated in the presence of IL2-ep13nsEV. (**G**) Activated and expanded spleenocytes from (**E**) were examined for antigen-specific priming by tetramer assay. Unpaired Student's t-test was used for data analysis ($n = 3$/group/ biological replicates). (**H**) B16-F1 cells were seeded in a 96-well plate. Bone marrow-derived dendritic cells (BMDCs) isolated from syngeneic mice were pulsed with B16-F1 lysate and treated with or without macbecin II. Subsequently, T cells isolated from the spleen of syngeneic mouse were co-cultured with the BMDCs for antigenic priming. T cells co-cultured with macbecin II-treated, lysate-pulsed BMDCs were designated as T+, while those co-cultured with macbecin II-untreated BMDCs were designated as T−. The primed T cells (E:T ratio 10:1) were then co-cultured with B16-F1 cells treated with or without macbecin II for 48 h. After incubation, dead cells and T cells were washed off with PBS, and the remaining cancer cells were fixed with methanol and stained with crystal violet. The dye was dissolved in 10% acetic acid, and absorbance was measured at 590 nm. Statistical significance between groups was determined using one-way ANOVA with Tukey's post-hoc test ($n = 3$/group, biological replicates). (**I**) MDA-MB-231 cells (1000 cells/well, $n = 3$/group, biological replicates) were cultured in the presence of in vitro educated PBMCs from Fig. 3A for 48 h. Post-incubation, cells were washed with ice-cold PBS to dislodge the dead cells and PBMCs. The live cells were fixed in methanol at room temperature for 15 min and stained with crystal violet. The dye was dissolved in 10% acetic acid and readouts were obtained at 590 nm. (**J**) E0771 cells (500 cells/well) were cultured in a 96-well plate and treated with (i) vehicle, (ii) macbecin II (0.1 μM and 0.5 μM), (iii) CD8 T cells isolated from the spleen of an E0771-bearing syngeneic mouse and activated with exosomes isolated from E0771 lysate-loaded BMDCs (50 μg/mL) for 5 days (E:T ratio 5:1 and 10:1) alone or in combination for 48 h ($n = 3$/group, biological replicates). After incubation, the cells were washed with ice-cold PBS to remove dead cells and T cells. The surviving cells were fixed in methanol and stained with crystal violet. The dye was dissolved in 10% acetic acid and readouts were obtained at 590 nm. (**K**) Combination index (CI) values were calculated by CompuSyn based on the inhibitory effect of macbecin II and T cell treatment. CI < 1 indicates synergy. (**L**) TNF-α expressions were measured for PBMCs that were treated with macbecin II. (**M**) Relative cell viability was assessed using the MTS assay in PBMCs that were treated with macbecin II. ($n = 3$/group, biological replicates). Data are represented as mean +/− SEM ($n = 5$/group, biological replicates).

© The Author(s)

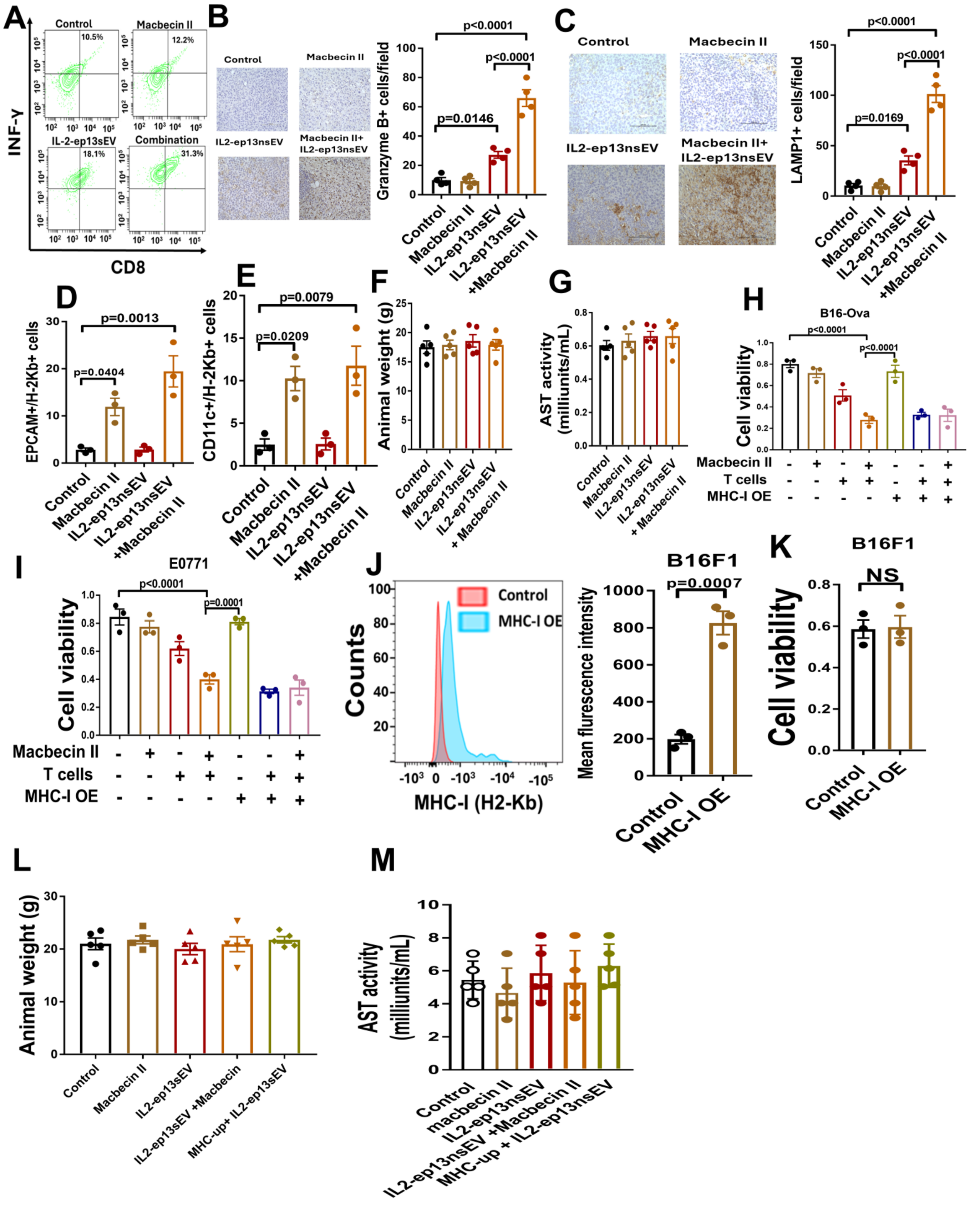
A
Control
Macbecin II
IL-2-ep13sEV
Combination
10.5%
12.2%
18.1%
31.3%
INF-γ
CD8
B
Control
Macbecin II
IL2-ep13nsEV
Macbecin II+
IL2-ep13nsEV
Granzyme B+ cells/field
p<0.0001
p<0.0001
p=0.0146
C
Control
Macbecin II
IL2-ep13nsEV
Macbecin II+
IL2-ep13nsEV
LAMP1+ cells/field
p<0.0001
p<0.0001
p=0.0169
Control
Macbecin II
IL2-ep13nsEV
IL2-ep13nsEV
+Macbecin II
D
EPCAM+/H-2Kb+ cells
p=0.0013
p=0.0404
E
CD11c+/H-2Kb+ cells
p=0.0079
p=0.0209
F
Animal weight (g)
G
AST activity
(milliunits/mL)
H
B16-Ova
Cell viability
p<0.0001
p<0.0001
Macbecin II
T cells
MHC-I OE
I
E0771
Cell viability
p<0.0001
p=0.0001
Macbecin II
T cells
MHC-I OE
J
Counts
Control
MHC-I OE
MHC-I (H2-Kb)
B16F1
p=0.0007
Mean flurescence intensity
Control
MHC-I OE
K
B16F1
NS
Cell viability
Control
MHC-I OE
L
Animal weight (g)
Control
Macbecin II
IL2-ep13sEV
IL2-ep13sEV +Macbecin
MHC-up+ IL2-ep13sEV
M
AST activity
(milliunits/mL)
Control
macbecin II
IL2-ep13nsEV
IL2-ep13nsEV +Macbecin II
MHC-up + IL2-ep13nsEV

© The Author(s)

Figure EV4. Macbecin II potentiates the anticancer efficacy of IL2-ep13nsEV in vivo.

(**A**) Flow cytometry analysis for INF-γ-CD8 + T cells in the four groups. (**B**) Granzyme B expression was examined by immunohistochemistry (left) and quantified (right) in the four groups. The staining intensity was analyzed by the unpaired two-tailed Student's t-test ($n = 4$/group, biological replicates). (**C**) LAMP1 expression was examined through immunohistochemistry (left) and quantified (right) in the four groups. The staining intensity was measured and analyzed by the one-way ANOVA with a Tukey post-hoc test ($n = 4$/group, biological replicates). (**D**, **E**) MHC-I expression was examined in EPCAM+ tumor cells and CD11c+ dendritic cells in the dissociated tumor by FACS. Statistical analysis was performed using the one-way ANOVA with a Tukey post-hoc test ($n = 3$/group, biological replicates). (**F**, **G**) Animal weight (**F**) and AST activity (**G**) in the blood of mice were measured at the end-point. Data are represented as mean +/− SEM ($n = 5$/group, biological replicates). (**H**) B16-Ova cells (500 cells/well, $n = 3$/group, biological replicates, were seeded in a 96-well plate and treated with a combination of macbecin II (0.1 μM) and OT-1 T cells (E:T ratio 5:1) with or without ectopic MHC-I expression for 48 h. The T cells were activated with IL2-ep13nsEV that were isolated from B16-Ova lysate-pulsed BMDCs (E:T ratio 5:1). After the incubation, cells were washed with ice-cold PBS to remove dead cells. The live cells were fixed with methanol at room temperature for 15 min and stained with crystal violet. The dye was dissolved in 10% acetic acid and measured at 590 nm. (**I**) E0771 cells (500 cells/well, $n = 3$/group, biological replicates) were seeded in a 96-well plate and treated with a combination of T cells (E:T ratio 5:1) and macbecin II (0.1 μM) with or without ectopic MHC-I expression for 48 h. The T cells were isolated from the spleen of E0771-bearing syngeneic mouse and they were activated with IL2-ep13nsEV that were isolated from E0771 lysate-pulsed BMDCs. After the incubation, cells were processed as described in (**H**). Statistical significance was determined by the one-way ANOVA with a Tukey post-hoc test. (**J**) MHC-I was ectopically expressed in B16-F1 cells, and the MHC-I expression was confirmed by FACS. The result was analyzed by the unpaired two-tailed Student's t-test ($n = 3$/group, biological replicates). (**K**) Cell viability was examined by MTS assay for B16-F1 with or without MHC-I over expression (OE) ($n = 3$/group, biological replicates). (**L**, **M**) Animal weight (**L**) and AST activity (**M**) were measured at the end-point ($n = 5$/group, biological replicates). Data are presented as mean +/− SEM.

© The Author(s)

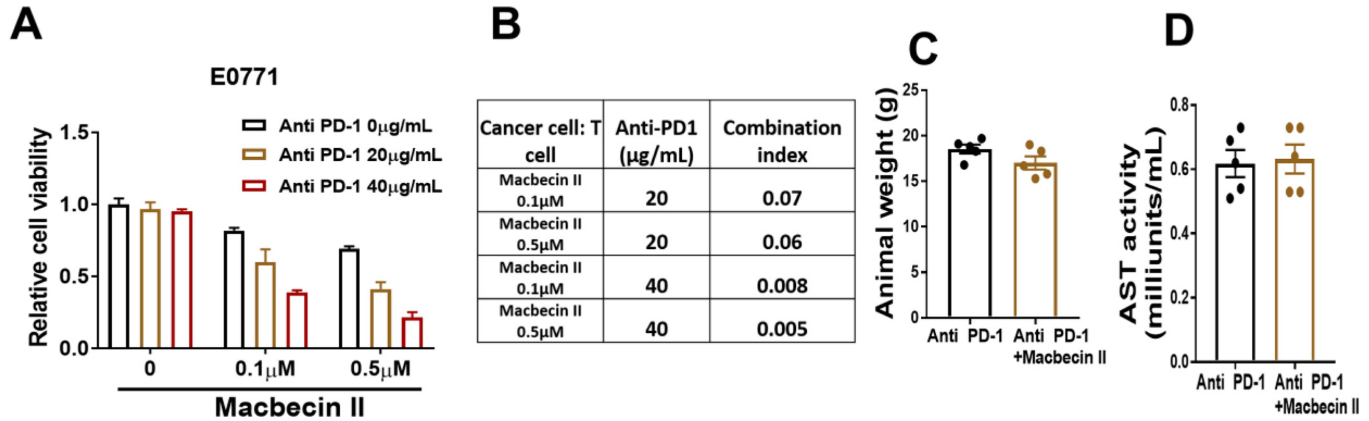

Cancer cell: T cell	Anti-PD1 (μg/mL)	Combination index
Macbecin II 0.1μM	20	0.07
Macbecin II 0.5μM	20	0.06
Macbecin II 0.1μM	40	0.008
Macbecin II 0.5μM	40	0.005

Figure EV5. Macbecin II potentiates anticancer efficacy of anti-PD-1 immune checkpoint blockade in breast cancer.

(**A**) E0771 cells (500 cells/well) were cultured in a 96-well plate in the presence of (i) vehicle, (ii) anti-PD-1 (20 μg and 40 μg), and (iii) Macbecin II (0.1 μM and 0.5 μM). CD8 T cells were isolated from the spleen of an E0771-bearing syngeneic mouse and activated with CD3/CD28 beads. The activated CD8 T cells were co-cultured with the E0771 cells ($n = 3$/group, biological replicates 5:1 E:T ratio). After 48 h, the cells were washed with ice-cold PBS to remove dead cells and T cells. The surviving cells were fixed in methanol and stained with crystal violet. The dye was dissolved in 10% acetic acid and readouts were obtained at 590 nm. (**B**) Combination index (CI) values were calculated by CompuSyn based on the inhibitory effect of anti-PD-1 and macbecin II treatment. CI < 1 indicates synergy. (**C**, **D**) Animal weight (**A**) and AST activity (**B**) were measured at the end-point ($n = 5$/group, biological replicates). Data are presented as mean +/− SEM.

 © The Author(s)

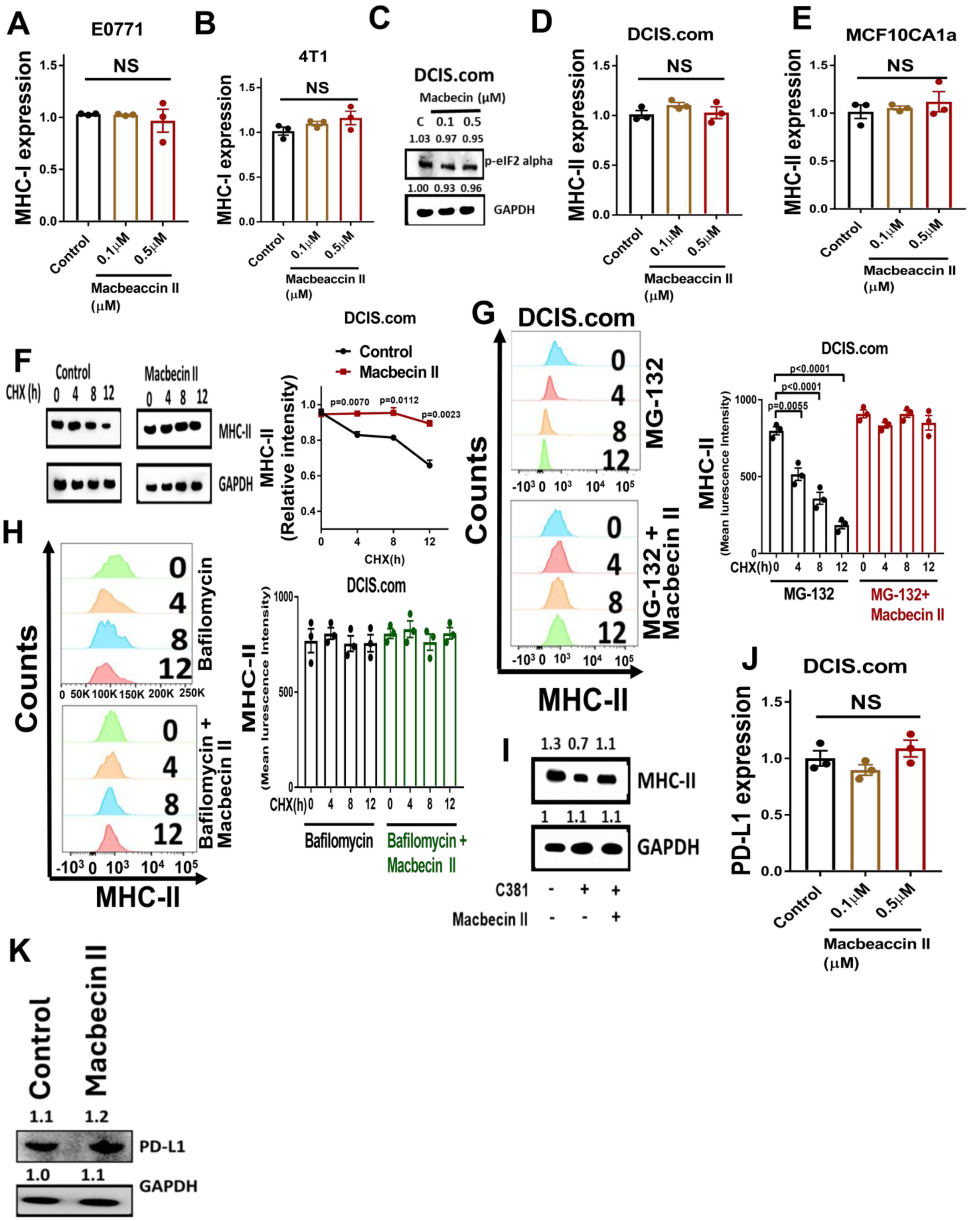
A
E0771
MHC-I expression
NS
Control
0.1μM
0.5μM
Macbeaccin II (μM)
B
4T1
MHC-I expression
NS
Macbeaccin II (μM)
C
DCIS.com
Macbecin (μM)
C 0.1 0.5
1.03 0.97 0.95
p-eIF2 alpha
1.00 0.93 0.96
GAPDH
D
DCIS.com
MHC-II expression
NS
Macbeaccin II (μM)
E
MCF10CA1a
MHC-II expression
NS
Macbeaccin II (μM)
F
Control
Macbecin II
CHX (h) 0 4 8 12
MHC-II
GAPDH
DCIS.com
Control
Macbecin II
p=0.0070
p=0.0112
p=0.0023
MHC-II (Relative intensity)
CHX(h)
G
DCIS.com
Counts
MG-132
MG-132 + Macbecin II
MHC-II
DCIS.com
p<0.0001
p<0.0001
p=0.0055
MHC-II (Mean lurescence Intensity)
CHX(h)
MG-132
MG-132+ Macbecin II
H
Counts
Bafilomycin
Bafilomycin + Macbecin II
MHC-II
DCIS.com
MHC-II (Mean lurescence Intensity)
CHX(h)
Bafilomycin
Bafilomycin + Macbecin II
I
1.3 0.7 1.1
MHC-II
1 1.1 1.1
GAPDH
C381 - + +
Macbecin II - - +
J
DCIS.com
PD-L1 expression
NS
Control
0.1μM
0.5μM
Macbeaccin II (μM)
K
Control
Macbecin II
1.1 1.2
PD-L1
1.0 1.1
GAPDH

Figure EV6. Macbecin II upregulates MHC-I and II through inhibition of lysosomal degradation.

(**A**, **B**) E0771 (**A**) and 4T1 (**B**) cells were seeded in a 24-well plate and treated with the indicated dose of macbecin II for 48 h. Total RNA was isolated, and cDNA synthesis was performed with the iScript cDNA Synthesis Kit as per the manufacturer's instructions followed by SYBR green-based Real Time PCR. GAPDH was used as an internal control. Statistical inference was determined by one-way ANOVA with a Tukey post-hoc test ($n = 3$/group, biological replicates). (**C**) DCIS.com cells were treated with the indicated dose of macbecin II for 48 h. Post-incubation, the total protein was isolated and p-eIF2 alpha expression was examined by western blot. Data are represented as mean +/− SEM. (**D**, **E**) DCIS.com (**D**) and MCF10CA1a (**E**) cells were seeded in 24-well plates and treated with the indicated doses of macbecin II for 48 h. Total RNA was isolated, and cDNA synthesis was performed using the iScript™ cDNA Synthesis Kit according to the manufacturer's instructions, followed by SYBR Green-based real-time PCR. GAPDH was used as the internal control. Statistical analysis was performed using the one-way ANOVA with a Tukey post-hoc test ($n = 3$/group, biological replicates). (**F**) DCIS.com cells were treated with cycloheximide (50 µg/ml) and macbecin II (0.5 µM) for the indicated times. MHC-II expression was examined by western blot (left panel) which was quantified using ImageJ (right panel) ($n = 3$/group, biological replicates). A two-tailed unpaired Student's t-test was used for analysis. (**G**) DCIS.com cells were treated with vehicle, a combination of MG-132 (10 µM) and macbecin II (0.5 µM), or MG-132 alone in the presence of cycloheximide (50 µg/ml) at the indicated time. MHC-II expression was examined by FACS, and the data was analyzed by the one-way ANOVA with a Tukey post-hoc test ($n = 3$/group, biological replicates). (**H**) DCIS.com cells were treated with vehicle, a combination of Bafilomycin (100 nM) and macbecin II (0.5 µM), or Bafilomycin alone in the presence of cycloheximide (50 µg/ml) at the indicated time. MHC-II expression was then examined by FACS, and the data was analyzed by the one-way ANOVA with a Tukey post-hoc test ($n = 3$/group, biological replicates). (**I**) DCIS.com cells were seeded in a 24-well plate and cultured in the presence of cycloheximide (50 µg/ml). Cells were treated with the vehicle, a combination of C381 (30 µM) and macbecin II (0.5 µM), or C381 alone for 12 h and MHC-II expression was examined by western blot. (**J**) DCIS.com cells were seeded in a 24-well plate and treated with the indicated doses of macbecin II for 48 h. Total RNA was isolated, and cDNA synthesis was performed using the iScript™ cDNA Synthesis Kit according to the manufacturer's instructions, followed by SYBR Green-based Real-Time PCR. GAPDH was used as the internal control. Statistical analysis was performed using the one-way ANOVA with a Tukey post-hoc test ($n = 3$/group, biological replicates). (**K**) DCIS.com cells were treated with macbecin II (0.5 µM), and PD-L1 expression was examined by western blot. Data are presented as mean +/− SEM.

© The Author(s)
